# Inhibition of Amyloid-Beta Production, Associated Neuroinflammation, and Histone Deacetylase 2-Mediated Epigenetic Modifications Prevent Neuropathology in Alzheimer’s Disease *in vitro* Model

**DOI:** 10.3389/fnagi.2019.00342

**Published:** 2020-01-15

**Authors:** Venkata Subba Rao Atluri, Sneham Tiwari, Melisa Rodriguez, Ajeet Kaushik, Adriana Yndart, Nagesh Kolishetti, Mohan Yatham, Madhavan Nair

**Affiliations:** ^1^Department of Immunology and Nano-Medicine, Institute of NeuroImmune Pharmacology, Herbert Wertheim College of Medicine, Florida International University, Miami, FL, United States; ^2^Division of Sciences, Art, & Mathematics, Department of Natural Sciences, Florida Polytechnic University, Lakeland, FL, United States

**Keywords:** amyloid-beta, Alzheimer’s disease, inflammation, withaferin A, cytokine release inhibitory drug 3, mithramycin A

## Abstract

Alzheimer’s disease (AD) is a growing global threat to healthcare in the aging population. In the USA alone, it is estimated that one in nine persons over the age of 65 years is living with AD. The pathology is marked by the accumulation of amyloid-beta (Aβ) deposition in the brain, which is further enhanced by the neuroinflammatory process. Nucleotide-binding oligomerization domain, leucine rich repeat and pyrin domain containing 3 (NLRP3) and nuclear factor kappa-light-chain-enhancer of activated B cells (NF-κB) are the major neuroinflammatory pathways that intensify AD pathogenesis. Histone deacetylase 2 (HDAC2)-mediated epigenetic mechanisms play a major role in the genesis and neuropathology of AD. Therefore, therapeutic drugs, which can target Aβ production, NLRP3 activation, and HDAC2 levels, may play a major role in reducing Aβ levels and the prevention of associated neuropathology of AD. In this study, we demonstrate that withaferin A (WA), an extract from *Withania somnifera* plant, significantly inhibits the Aβ production and NF-κB associated neuroinflammatory molecules’ gene expression. Furthermore, we demonstrate that cytokine release inhibitory drug 3 (CRID3), an inhibitor of NLRP3, significantly prevents inflammasome-mediated gene expression in our *in vitro* AD model system. We have also observed that mithramycin A (MTM), an HDAC2 inhibitor, significantly upregulated the synaptic plasticity gene expression and downregulated HDAC2 in SH-SY5Y cells overexpressing amyloid precursor protein (SH-APP cells). Therefore, the introduction of these agents targeting Aβ production, NLRP3-mediated neuroinflammation, and HDAC2 levels will have a translational significance in the prevention of neuroinflammation and associated neurodegeneration in AD patients.

## Introduction

Alzheimer’s disease (AD), the most prevailing cause of dementia in the world, affects nearly 5.7 million Americans. The symptoms include dysfunctions in memory, difficulties in everyday behaviors, and cognitive impairment. Excessive deposition of amyloid-beta (Aβ), Tau hyperphosphorylation, and neuroinflammation are the hallmarks of AD. Neuroinflammation is one of the important pathological features in the progression of AD and associated neurocognitive impairment. Neuroinflammation is stimulated by damaged neurons and deposition of insoluble Aβ peptide and neurofibrillary tangles (NFTs) (Neuroinflammation Working Group et al., 2000). Microglia, the phagocytes of the central nervous system, are one of the key players in the maintenance and plasticity of neuronal circuits and are also helpful in protecting and remodeling synapses. In AD, microglia are stimulated by binding to soluble Aβ oligomers and Aβ fibrils via cell-surface receptors, which induce inflammatory reaction by the activation of nucleotide-binding oligomerization domain, leucine rich repeat and pyrin domain containing 3 (NLRP3) inflammasomes and nuclear factor kappa-light-chain-enhancer of activated B cells (NF-κB) pathway resulting in proinflammatory cytokines and chemokines release (Bonaiuto et al., 1997; Agostinho et al., 2010; Cameron and Landreth, 2010; Mandrekar-Colucci and Landreth, 2010; Heneka et al., 2013). Microglia engulf Aβ fibrils by phagocytosis and degrade the fibrils using proteases like neprilysin and insulin-degrading enzyme. In AD patients, activation of NLRP3 and NF-κB cascades inhibit the Aβ phagocytosis by microglia leading to enhanced deposition of Aβ fibrils, thereby creating a self-perpetuating loop, which further induces the neuroinflammation (Heneka et al., 2013).

NF-κB is a major transcription factor and plays an important role in the inflammatory response. NF-κB-mediated inflammatory response is a common feature of many neurodegenerative diseases such as Huntington, Parkinson’s, stroke, and particularly AD (Boissière et al., 1997; Kaltschmidt et al., 1997; Lukiw and Bazan, 1998). In the brain of AD patients, NF-κB activation is predominantly found in neurons and glial cells present in the vicinity of Aβ plaque deposition (Terai et al., 1996; Boissière et al., 1997; Kaltschmidt et al., 1997; Lukiw and Bazan, 1998). In our earlier study, we have observed that withaferin A (WA), a steroidal lactone, derived from the plant *Withania somnifera* inhibits Aβ levels in amyloid overexpressing SH-SY5Y cells (SH-APP) (Tiwari et al., 2018). Based on these observations and reports on the role of WA in inhibiting the NF-κB-mediated neuroinflammation (Heyninck et al., 2014; Martorana et al., 2015), in this study we have utilized WA to study the Aβ levels and associated NF-κB-mediated neuroinflammation in SH-APP and microglial mixed cell culture.

The NLRP3 inflammasome, which can sense inflammatory crystals and aggregated proteins including Aβ, has been recently implicated in several chronic inflammatory diseases (Halle et al., 2008; Martinon et al., 2009). In response to the chronic Aβ deposition, in AD, microglial cells are persistently activated (Prinz et al., 2011) and result in increased interleukin-1β (IL-1β) levels (Lucin and Wyss-Coray, 2009). For the conversion of inactive pro-form of IL-1β to mature form, caspase-1 is required and caspase-1 activity is controlled by the inflammasomes. Furthermore, the increase of caspase-1 processing has been observed in aged APP/PS1 transgenic mice (Heneka et al., 2013). Therefore, along with the inhibition of NF-κB-mediated inflammatory response, inhibition of NLRP3-mediated inflammatory response is required for the complete prevention of inflammatory response in AD patients. Recently, cytokine release inhibitory drugs (CP-456,773/CRID3, CP-424,174, and CP-412,245) have been identified as novel inhibitors of NLRP activation and subsequent IL-1β production (Coll et al., 2015). In this study, for the first time, we have explored the use of CRID3 in inhibiting inflammasome-mediated neuroinflammation in an *in vitro* AD model (SH-APP cells co-cultured with microglial CHME5 cells).

In addition, epigenetic mechanisms mediated by histone modifications are one of the major neuropathogenic mechanisms in AD. Within the mammalian nervous system, histone-modifying enzyme, histone deacetylase 2 (HDAC2) is a critical negative regulator of structural and functional plasticity. The HDAC2 deacetylates histone substrates at the promoter area of numerous synaptic-plasticity-associated genes (Guan et al., 2009; Gräff et al., 2012). Notably, both AD patient brains and multiple mouse models of AD have elevated levels of HDAC2 (Gonzalez-Zuñiga et al., 2014; Liu et al., 2017). Mithramycin A (MTM) is a gene selective specificity protein 1 (Sp1) inhibitor that is employed as a chemotherapeutic agent that inhibits tumor cell growth without affecting normal cells (Torrance et al., 2001). Its neuroprotective role, in case of Huntington’s disease has been studied in *in vitro* and *in vivo* experiments (Chatterjee et al., 2001; Ferrante et al., 2004; Qiu et al., 2006; Ryu et al., 2006; Voisine et al., 2007). Further, MTM has been reported to inhibit class I HDACs, specifically HDAC2 and 3 (Sleiman et al., 2011). Therefore, in this study, for the first time, we have used MTM to study its effect in inhibiting HDAC2 expression and in recovering neuronal plasticity gene expression in an AD cell culture model.

## Results

### Cytotoxicity of Withaferin A, Cytokine Release Inhibitory Drug 3, and Mithramycin A

**FIGURE 1:**
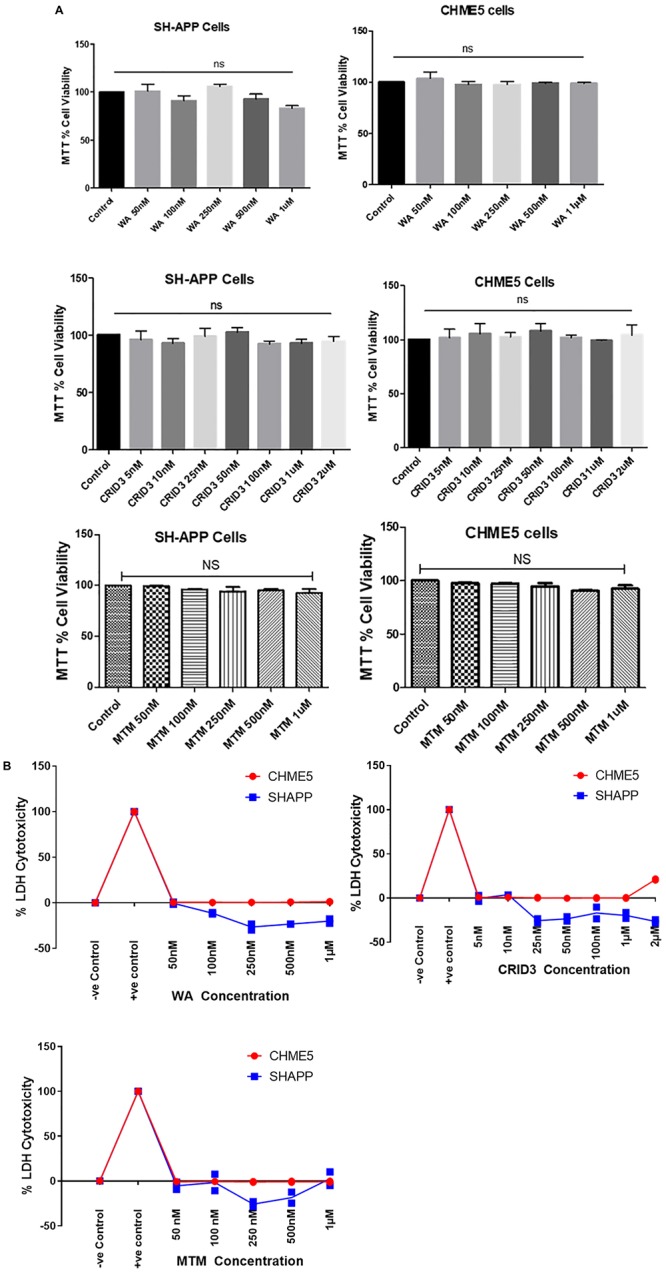
**(A)** MTT cell viability assay. Amyloid precursor protein overexpressing neuronal cells (SH-APP) and microglial cells were exposed to different concentrations of withaferin A (WA) (50 nM–1 μM) and cytokine release inhibitory drug (CRID3) (5 nM–2 μM). After 24 h of incubation, cell viability was analyzed by using MTT assay. We have observed no significant cell death with WA up to 1 μM and with CRID3 up to 2 μM in both the cell types. SH-APP cells were exposed to mithramycin A (MTM, 50 nM–1 μM) for 24 h and in MTT assay, we have not observed any significant cell death up to 1 μM concentration. **(B)** LDH cytotoxicity assay. Amyloid precursor protein overexpressing neuronal cells (SH-APP) and microglial cells were exposed to different concentrations of WA (50 nM–1 μM) and CRID3 (5 nM–2 μM). After 24 h of incubation, cell cytotoxicity was analyzed by using the LDH cytotoxicity assay. We have observed no cytotoxicity with WA up to 1 μM and with CRID3 up to 2 μM in both the cell types. SH-APP cells were exposed to MTM (50 nM–1 μM) for 24 h and we have not observed any cytotoxicity up to 1 μM concentration.

Using both MTT and LDH cytotoxicity assays, as shown in Figures 1A,B, respectively, we did not find significant cytotoxicity with the concentrations we have used for WA, CRID3, and MTM in both SH-APP and CHME5 cells.

### Congo Red Stain-Based Quantification of Amyloid-Beta in Withaferin A-Treated Cells

**FIGURE 2:**
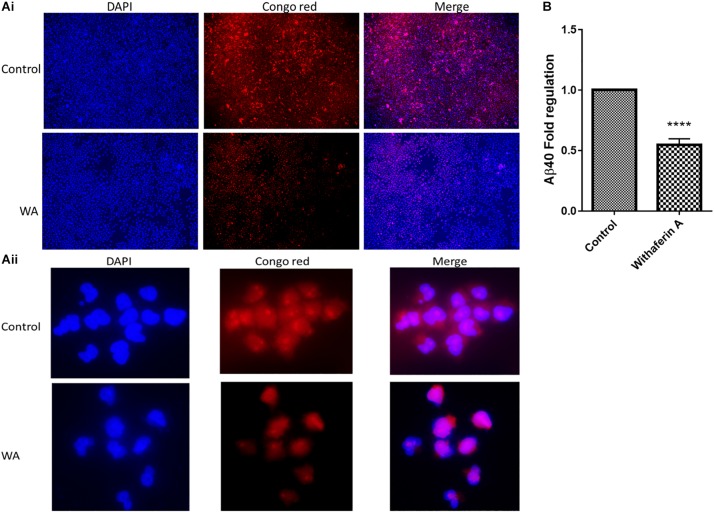
Congo red staining and amyloid-beta (Aβ40) ELISA for Aβ measurement: **(Ai,ii)** SH-APP cells were treated with 1 μM concentration of withaferin A and incubated for 48 h. Cells were stained with the Congo red stain. We have observed significantly reduced levels of amyloid-beta expression in WA-treated cells when compared to the DMSO-treated control cells. **(B)** SH-APP cells were cultured in six-well culture plates and after 48 h of incubation, cells were treated with withaferin A (1 μM). After 48 h of incubation, cell culture supernatant was analyzed for Aβ levels using Aβ40 ELISA and observed a significant reduction of Aβ40 levels upon WA treatment when compared to the DMSO-treated cells (^****^p ≤ 0.0001).

We studied the effect of WA on Aβ production by staining the cells with Congo red (CR) stain. As Figure 2A shows, SH-APP cell cultures treated with WA alone showed less staining with the toxic Aβ peptide than in dimethyl sulfoxide (DMSO)-treated SH-APP cell controls. CR tagged the Aβ peptide and stained them red in DMSO-treated control SH-APP cells expressing higher Aβ levels. Cells treated with WA had reduced levels of Aβ and therefore less staining was observed in these treatment group cells. We observed these cells at 10× resolution. This study aligns with our prior results that WA has the ability to reduce Aβ *in vitro* (Tiwari et al., 2018).

### Amyloid-Beta Quantification by ELISA

In support of the CR staining results, in the ELISA, we have quantitatively observed that WA inhibits the Aβ40 secretion in Aβ-overexpressing SH-APP cells (Figure 2B).

### Withaferin A Inhibits Inflammatory Regulated Genes Associated With the Nuclear Factor-κB Pathway

SH-APP cells and CHME5 microglia cell lines were co-cultured for 48 h and incubated with WA. After 48 h of treatment, cells were analyzed for the NF-κB-mediated inflammatory response mediators using the human NF-κB Signaling Pathway PCR Array. We observed that WA inhibited the expression of NF-κB subunit 2 (NFκB2) and RELA transcription factors which play a major role in the expression of inflammatory chemokines and cytokines. We also observed the IKBKB and IKBKG upregulation (depletion of these proteins activate the NF-κB) and JUN and STAT genes’ downregulation. Furthermore, we have observed downregulation of IL-1β which plays a major role in the NF-κB mediated neuroinflammation (Table 1).

**TABLE 1:** Withaferin A (WA) inhibits nuclear factor-kappa B (NF-κB)-mediated inflammatory genes expression.

**Genes**	**WA (fold change)**
*1L1B*	–4.0558
*IL-10*	4.69
*NFKB1*	1.6818
*NFKB2*	–1.2834
*REL*	1.3755
*RELA*	–1.021
*RELB*	1.2746
*BCL3*	–1.2226
*CHUK*	–1.1975
*IKBKB*	1.4743
*IKBKG*	2.1287
*NFKBIA*	–1.014
*NFKBIE*	1.3287
*JUN*	–1.181
*STAT1*	–1.0281

*Human microglia and SH-APP cells were co-cultured and exposed to WA. After 48 h of incubation, using human NF-κB Signaling Pathway PCR Array, we observed significant down-regulation of transcription factors; pro-inflammatory cytokines and apoptosis-inducing genes expression in the culture indicate the NF-κB-mediated anti-inflammatory activity of WA in AD cell culture model. (*p* ≤ 0.05 for all the genes listed in the table).*

### Cytokine Release Inhibitory Drug 3 Inhibits Inflammasome Regulated Gene Expression

In this study, SH-APP and CHME5 microglia cell lines were co-cultured and exposed to 100 nM CRID3. After 48 h of incubation, cells were harvested, RNA was isolated, and human inflammasome PCR array was performed. Results demonstrated that CRID3 significantly inhibited various chemokines and inflammasome regulated gene expression (Table 2). We observed significant downregulation of Caspase 1 gene expression, which activates the release of mature IL-1β. Our results indicated reduction in IRF2 levels, and reduced IRF2 may hamper gasdermin-D (GSDMD) expression, which results in reduced IL-1β secretion and inhibits pyroptosis (Kayagaki et al., 2019). CRID3 also inhibited RELA gene, which is a subunit of the transcriptional factor NF-κB. Furthermore, we observed the inhibition of RIPK2, which is a potent activator of NF-κB and inducer of apoptosis. In addition, we observed the downregulation of TRAF6 gene expression, which may inhibit caspase-1 cleavage, pyroptosis, and pre-synthesized IL-18 secretion (Xing et al., 2017). Upregulation of IL-6 and TXNIP genes upon CRID3 exposure may help in reducing oxidative stress and inflammatory response.

**TABLE 2:** Cytokine release inhibitory drug (CRID3) inhibits inflammasome associated genes expression.

**Genes**	**CRID3 (fold change)**
*CASP1*	–2.0705
*CCL5*	–2.6945
*CCL7*	2.6574
*IRF2*	–3.8906
*RELA*	–2.514
*RIPK2*	–2.1585
*TRAF6*	–3.2043
*IKBKB*	1.3755
*NFKB1*	–1.1892
*IL-6*	2.1585
*TXNIP*	2.0705

*Human microglia and AβPP overexpressing cells were co-cultured and exposed to CRID3 (100 nM). After 48 h of incubation, using human inflammasome genes PCR array, we observed down-regulation of inflammasome forming genes expression in these cells indicating the anti-inflammasome activity of CRID3 in AD cell culture model (*p* ≤ 0.05 for all the genes listed in the table).*

### Cytokine Release Inhibitory Drug 3 Inhibits Caspase-1 and IL-1β Protein Expression

**FIGURE 3:**
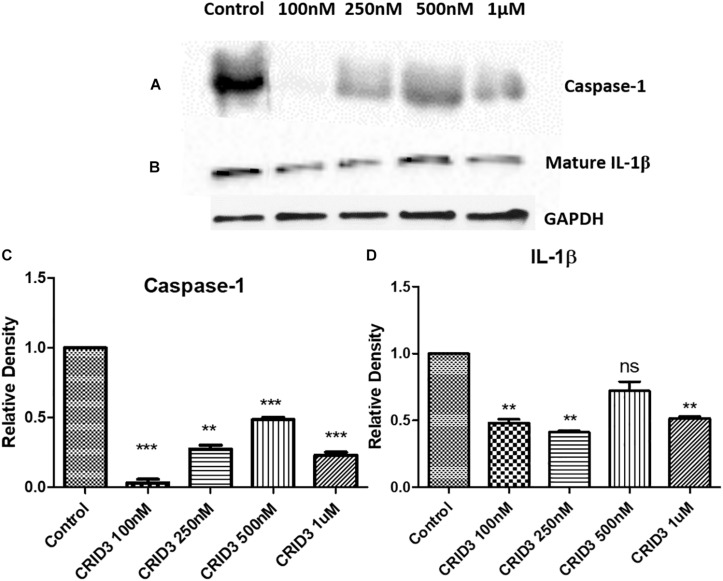
Cytokine release inhibitory drug (CRID3) inhibits Caspase-1 and IL-1β protein expression. SH-APP cells were co-cultured with CHME5 microglia cell line (microglia: neuron ratio of 1:2) in the presence of CRID3 (25–100 nM) for 48 h. Cells were harvested and the cell pellet was used in western blot for Caspase-1 **(A)** and IL-1β **(B)** protein expression analysis. Relative protein expression was calculated using ImageJ and a significant inhibition was observed for Caspase-1 **(C)** and IL-1β **(D)** protein expression at 100 nM CRID3 concentration compared to the water-treated control cells (*n* = 3) (^∗∗^*p* ≤ 0.01; ^∗∗∗^*p* ≤ 0.001).

Caspase-1 (IL-1 converting enzyme) and consequently released mature IL-1β production plays a major role in the neuroinflammatory response in the AD patients. In this study, we have used various concentrations of CRID3 (25–100 nM) to see its efficacy in inhibiting caspase-1 activation and IL-1β production in SH-APP cells co-cultured with CHME5 microglia cell line (microglia: neuron ratio of 1:2). Mixed cell culture was incubated for 48 h in the presence of the CRID3. Cells were harvested, centrifuged, and protein was isolated from the cell pellet. We analyzed the expression of caspase-1 and mature IL-1β protein expression, by quantifying via Western blot assay. Our data demonstrates the significant down-regulation of caspase-1 and mature IL-1β protein levels at 100 nM CRID3 concentration (Figure 3) indicating its potential therapeutic role in the prevention of neuronal apoptosis due to the accumulation of Aβ in AD patients.

### Mithramycin A Inhibits Histone Deacetylase 2 Expression

**FIGURE 4:**
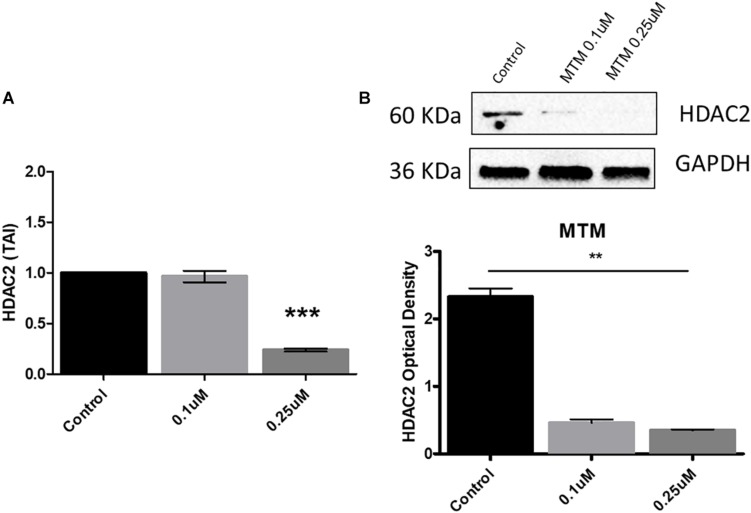
Mithramycin A (MTM) inhibits histone deacetylase 2 (HDAC2) expression. Amyloid overexpressing cells (SH-APP) were exposed to different concentrations of MTM and found that at 0.25 μM concentration significantly inhibits both HDAC2 gene **(A)** and protein **(B)** expression (^∗∗^*p* ≤ 0.01; ^∗∗∗^*p* ≤ 0.001).

We demonstrate that MTM significantly inhibits the HDAC2 gene and protein expression at 0.25 μM concentration in Aβ overexpressing SH-APP cells (Figures 4A,B).

### Mithramycin A Improves Neuronal Plasticity Genes Expression in Amyloid Precursor Protein-Overexpressing Cells

SH-APP cells were treated with MTM and incubated for 48 h. RNA was isolated from the harvested cells, and the first-strand cDNA was synthesized using the standard protocol. Human synaptic plasticity genes PCR array was performed, and out of 84 synaptic plasticity genes we analyzed, 15 genes were significantly up-regulated (Table 3). These genes play a major role in neuroprotection and long-term memory formation. Importantly, an upregulated ARC gene is required for protein synthesis-dependent forms of long-term potentiation (LTP) and long-term depression (LTD) and the formation of long-term memory. Nerve growth factor (NGF) and NGFR are neuroprotective agents involved in nerve growth stimulating activity. Upregulation of c-Jun/FOS and related transcription factors aid adult brain functions and injury repair. The TIMP1 are natural inhibitors of matrix metalloproteinases (MMP), induce cell proliferation, and have anti-apoptotic activity. Furthermore, we did not observe the down-regulation of any neuronal plasticity genes measured in the array.

**TABLE 3:** Human synaptic plasticity genes expression in mithramycin A-treated SH-APP cells.

**Synaptic plasticity genes**	**Fold up-regulation**
*ARC*	8.3109
*CDH2*	2.3054
*CEBPD*	4.9075
*EGR2*	9.4153
*FOS*	4.1843
*GRIN2C*	3.1492
*JUNB*	9.68
*KLF10*	3.5925
*NGF*	2.2423
*NGFR*	7.8626
*NOS1*	3.6175
*PIM1*	2.7606
*RGS2*	5.7557
*TIMP1*	5.7557
*TNF*	4.5159

*Using human synaptic plasticity genes PCR array analysis, we observed significant up-regulation of 15 synaptic plasticity genes, which play a major role in neuroprotection and long-term memory formation. None of the synaptic plasticity genes were significantly down-regulated upon MTM exposure (*p* ≤ 0.05 for all the genes listed in the table).*

## Discussion

AD is the most common dementia with no accountable universally accepted theory for the associated risk factors, leading to the development of pathological and clinical features. Failure of Phase III clinical trial using anti-amyloid antibody warrants to focus on alternate pathological mechanisms. Increasing evidence suggests that AD pathogenesis is not only limited to the production of misfolded or aggregated Aβ and Tau-hyperphosphorylation but also depends on the innate immune response through activation of microglia/astrocytes leading to the activation of inflammatory pathways that induce neuronal apoptosis in response to the excessive Aβ production (Heneka et al., 2015; Calsolaro and Edison, 2016; Ardura-Fabregat et al., 2017).

Several studies have shown that Aβ and/or a secreted form of the amyloid precursor protein (AβPP) induces the upregulation of NF-κB activity (Bonaiuto et al., 1997). Both pathological features of AD (Aβ and Tau hyperphosphorylation) are capable of inducing NF-κB activation via various mechanisms. One common mechanism is the activation of the advanced glycation end products or receptor for advanced glycation end products (AGE/RAGE) signaling pathway. Through the non-enzymatic glycation, Aβ and Tau can form AGEs and these AGEs bind to RAGEs and trigger NF-κB-dependent gene transcription and translocation of NF-κB to the nucleus (Stern, 1995). Therefore, in this study, we have targeted the Aβ production and associated NF-κB activation using the WA and observed reduced Aβ levels in amyloid overexpressing SH-APP cells and NF-κB-mediated inflammatory gene expression.

Innate immune activation, in response to the pattern recognition receptors’ recognition to the misfolded or aberrant proteins (Aβ in case of AD) (Barger and Harmon, 1997; Meda et al., 1999), results in the activation of NLRP3 components in the inflammasome formation (Schnaars et al., 2013; Nakanishi et al., 2018; Yin et al., 2018). Caspase-1 activity is controlled by inflammasomes (Franchi et al., 2009) which in turn activates the conversion of pro-IL-β into mature IL-β. Excessive mature IL-1β production induces neuroinflammation, Tau-hyperphosphorylation, neuronal and synaptic dysfunctions, and Aβ plaque accumulation (Griffin et al., 1998, 2006; Sheng et al., 2000). In this study, with the use of novel inflammasome inhibitor CRID3, we have demonstrated the inhibition of multiple inflammasomes associated genes expression as well as decreased caspase-1 and IL-1β levels in an *in vitro* AD cell culture model.

Furthermore, since within the mammalian nervous system, histone-modifying enzyme HDAC2 is a critical negative regulator of structural and functional plasticity, when this enzyme localizes to promoters, it deacetylates histone substrates of numerous synaptic-plasticity-associated genes (Guan et al., 2009; Gräff et al., 2012). Elevated levels of HDAC2 have been found in AD patient’s brains and multiple mouse models of AD (Gonzalez-Zuñiga et al., 2014; Liu et al., 2017). In this study, for the first time, we demonstrate that MTM, a sp1 inhibitor, inhibits HDAC2 upregulation and achieves significantly reduced HDAC2 gene and protein expression resulting in the recovery of synaptic plasticity genes expression in SH-APP cells. In addition, other experimental evidences demonstrate that HDAC inhibitors improve cognitive ability and aid in re-establishing long-term memory (Kazantsev and Thompson, 2008; Guan et al., 2009). In murine model experiments, treatment with sodium butyrate, an HDAC inhibitor, has exhibited restoration of learning ability and long-term memory despite significant neuronal loss (Fischer et al., 2007). Therefore, the identification of neuronal plasticity and cognitive functions regulating HDAC(s) could lead to the development of target-specific therapies. Studies (Guan et al., 2009) reported that HDAC2 expression is inversely related to neuronal plasticity, memory formation, and neuronal spine density/neuronal morphology associated with the memory. Specifically, HDAC2 associates with the negative regulation of neuronal plasticity genes and memory formation (Levenson et al., 2004; Guan et al., 2009). Furthermore, studies using HDAC inhibitors in mice with conditional alleles to HDAC1 and HDAC2 confirmed the role of HDAC2 in synapse maturation and regulation of neurons (Akhtar et al., 2009). Therefore, the use of MTM in AD patients may help in restoring neuronal plasticity and memory.

## Conclusion

Excessive Aβ production and associated neuroinflammation and epigenetic modifications contribute to the altered neuronal plasticity and loss of memory in AD patients. Therefore, the use of small molecule drugs, WA and CRID3, will have a translational significance in inhibiting Aβ production and associated NF-κB and inflammasome-mediated neuroinflammation. Epigenetic modifications mediated by HDAC2 upregulation can be targeted by MTM. Further, preclinical studies using the combination of these therapeutic drugs need to be investigated both in *in vitro* and *in vivo* to validate their efficacy in inhibiting neuropathology of AD.

## Materials and Methods

### Cells and Reagents

SH-SY5Y cells transfected with amyloid precursor protein plasmid (SH-APP) were obtained as a gift from Dr. Jonathan Geiger, University of North Dakota. SH-SY5Y cells were cultured in equal parts of (1:1) DMEM and F-12 media (ScienCell, Catalog No. 09321). Immortalized microglial cells (CHME5) and SH-APP cells were maintained in Dulbecco’s Modified Eagle Medium (ScienCell, Catalog No. 09211) supplemented with the fetal bovine serum to a final concentration of 10 and 1% antibiotic/antimycotic solution. WA was commercially purchased from Sigma-Aldrich (Cat. No. W4394). CRID3 (Cat. No. 5479) and MTM (Cat. No. 1489) were purchased from Tocris, Minneapolis, MN, United States.

### Cytotoxicity of Withaferin A, Cytokine Release Inhibitory Drug 3, and Mithramycin A

SH-APP and CHME5 cells were grown in 96-well plates (20,000 cells/well) and incubated for 24 h. Cells were treated with different concentrations of WA (50 nM–1 μM), CRID3 (5 nM–2 μM), and MTM (50 nM–1 μM), and after 24 h of incubation, the MTT and LDH cytotoxicity assays were performed by following the kit protocol (Invitrogen CyQUANT LDH Cytotoxicity Assay, Cat. No. C20300).

For the MTT assay, after 24 h of treatment with WA, CRID3, and MTM as described above, the culture media was exchanged with 100 μl of fresh medium and 10 μl MTT (0.5% MTT in PBS) in each well and incubated at 37°C for 2–3 h. Following this, one volume of 110 μl of stop solution was added and the plates were rocked for approximately 2–3 h. The solubilized formazan optical density was measured spectrophotometrically at 550 nm. The OD of formazan in each well is directly proportional to the cell viability and was utilized for calculations.

### Congo Red Staining for Staining Amyloid-Beta

SH-APP cells were grown in two chamber slides at a concentration of 5.0 × 10^3^/ml for 48 h. The cells were then treated with an optimized concentration of WA (1 μM) for a further 48 h. WA was dissolved in DMSO and used at a final concentration of 1 μM. Control cultures were treated with DMSO (the same concentration used in the treatment group). After 48 h, the cell culture supernatant was discarded, and cells were washed with PBS followed by fixation with 4% formalin at room temperature for 15 min. Subsequently, cells were washed again with PBS and stained with 0.5% filtered CR stain (Sigma-Aldrich), at room temperature for 5 min. The cells were then washed with deionized water carefully, and the slides were mounted in DAPI Fluoromount-G^®^ (Southern Biotech, Catalog No. 0100-20) and then observed through Keyence microscope. The images were captured at a magnification of 10×.

### Amyloid-Beta (Aβ40) Quantification by ELISA

Amyloid-beta overexpressing SH-APP cells were grown in six-well culture plates and allowed to grow for 48 h. These cells were treated with WA (1 μM) and further incubated for 48 h. Cell culture supernatant was collected and analyzed for extracellular Aβ40 levels using the Aβ40 ELISA kit (Thermo Fisher Scientific, Catalog No. KHB3481).

### Estimation of Nuclear Factor-κB-Mediated Neuroinflammatory Genes Expression on Withaferin A Treatment

SH-APP cells and microglial cells (CHME5) were grown (2:1 ratio) in six-well plates for 48 h and treated with the optimized concentration of WA and incubated for further 48 h. The cell culture supernatant was discarded, and cells were collected and pelleted. RNA was collected from these cells using the standard protocol (Qiagen Cat. No. 74134). For the first-strand cDNA synthesis, 1 μg of RNA was used in SABiosciences’s RT2 First Strand Kit (Cat. No. 330401) as per supplier’s protocol. Before going for reverse transcription, genomic DNA elimination step was performed. RT^2^ Profiler^TM^ PCR Array Human NF-κB Signaling Pathway kit (Qiagen Cat. No. PAHS-025ZA-12) was used to measure 84 key genes associated with NF-κB-mediated signal transduction. This includes genes that encode members of the NFκB, Rel, and IkB families, NFκB-responsive genes, kinases, and transcription factors that propagate the signal and extracellular ligands and receptors that activate the pathway.

### Treatment With Cytokine Release Inhibitory Drug 3 and Estimation of Caspase-1 and IL-1β Levels

SH-APP cells and microglial cells (CHME5) were grown (2:1 ratio) in six-well plates for 48 h and treated with an optimized concentration of CRID3 and incubated for further 48 h. The cell culture supernatant was discarded, and cells were collected and pelleted. Protein and RNA were collected from these cells using the standard protocol. Protein samples were used to measure caspase-1 and mature IL-1β levels by the western blot assay using anti-caspase-1 (Cell Signaling Cat. No. 2225S) and anti-cleaved IL-1β antibody (Cell Signaling Cat. No. 83186S), respectively.

### Estimation of Inflammasome-Mediated Genes Expression on Cytokine Release Inhibitory Drug 3 Treatment

From CRID3-treated cells, RNA was collected using the standard kit protocol (Qiagen Cat. No. 74134). For the first-strand cDNA synthesis using SABiosciences’s RT2 First Strand Kit (Cat. No. 330401), 1 μg of RNA was used as per supplier’s protocol. Before going for reverse transcription, genomic DNA elimination step was performed. The human inflammasomes RT^2^ Profiler PCR Array (Qiagen Cat. No. PAHS-097Z) was then used to estimate the possible expression of the 84 key genes involved in inflammasomes function, innate immunity protein complexes, as well as general NOD-like receptor (NLR) signaling.

### Treatment With Mithramycin A and Estimation of Histone Deacetylase 2 Expression Levels

SH-APP cells were grown in six-well plates for 48 h and treated with an optimized concentration of MTM and incubated for further 48 h. The cell culture supernatant was discarded, and cells were collected and pelleted. Protein and RNA were collected from these cells using the standard protocol. Protein samples were used to measure HDAC2 levels by the western blot assay using anti-HDAC2 antibodies (Cell Signaling Cat. No. 2540S). RNA samples were analyzed for HDAC2 gene expression by qPCR.

### Human Synaptic Plasticity PCR Array on Mithramycin A Treatment

From MTM-treated SH-APP cells, RNA was collected using the standard kit protocol (Qiagen Cat. No. 74134). One microgram of RNA was used in SABiosciences’s RT2 First Strand Kit (Cat. No. 330401) as per supplier’s protocol. Before going for reverse transcription, genomic DNA elimination step was performed. To measure the expression of the 84 key genes essential to synaptic modifications during learning and memory, a Human Synaptic Plasticity RT^2^ Profiler PCR Array (Qiagen Cat. No. PAHS-126ZA) was utilized.

### Data Analysis

Experiments were performed as three independent biological sets and the data obtained was averaged. Using the ImageJ software, the relative densities of the detected protein bands in western blots were calculated and analyzed. Results were expressed as mean ± s.e.m. Student’s *t*-test was used for statistical analysis of two groups, while the one-way ANOVA was used to analyze any comparison of data between two or more groups, followed by Bonferroni’s multiple comparison tests. Differences were considered significant at *p* ≤ 0.05. GraphPad Prism software (La Jolla, CA, United States) was used for the data analysis.

## Data Availability Statement

The datasets generated for this study are available on request to the corresponding author.

## Author Contributions

VA and MN conceived and designed the experiments, and contributed reagents and materials. VA, ST, and MR performed the experiments. VA, ST, AK, AY, NK, and MY analyzed the data. VA and ST prepared the manuscript.

## Conflict of Interest

The authors declare that the research was conducted in the absence of any commercial or financial relationships that could be construed as a potential conflict of interest.
